# Modulation of NETosis in Swine Neutrophil–Spermatozoa Co-Cultures In Vitro: Effects of Butylated Hydroxytoluene, Albumin, Prostaglandin E_2_, and Seminal Plasma

**DOI:** 10.3390/antiox14070778

**Published:** 2025-06-24

**Authors:** Fabiola Zambrano, Felipe Pezo, André Furugen Cesar de Andrade, Rodrigo Rivera-Concha, Pamela Uribe, Mabel Schulz, Henricco Zapparoli, Luan Mendes de Oliveira Bezerra, Carlos Hermosilla, Anja Taubert, Raúl Sánchez

**Affiliations:** 1 Center of Excellence in Translational Medicine—Scientific and Technological Bioresource Nucleus (CEMT—BIOREN), Faculty of Medicine, Universidad de La Frontera, Temuco 4780000, Chile; fabiola.zambrano@ufrontera.cl (F.Z.); r.rivera07@ufromail.cl (R.R.-C.); pamela.uribe@ufrontera.cl (P.U.); mabel.schulz@ufrontera.cl (M.S.); 2 Department of Preclinical Sciences, Faculty of Medicine, Universidad de La Frontera, Temuco 4811230, Chile; 3 Facultad de Ciencias Agropecuarias y Medioambiente, Universidad de La Frontera, Temuco 4780000, Chile; felipe.pezo@ufrontera.cl; 4 Center of Excellence in Reproductive Biotechnology (CEBIOR-BIOREN), Faculty of Medicine, Universidad de La Frontera, Temuco 4780000, Chile; 5 Department of Animal Reproduction, School of Veterinary Medicine and Animal Science (FMVZ), University of São Paulo (USP), Pirassununga 13635-900, Brazil; andrefc@usp.br (A.F.C.d.A.); henricco.silva@usp.br (H.Z.); luan_mendes@usp.br (L.M.d.O.B.); 6 Ph.D. Program in Medical Sciences, Faculty of Medicine, Universidad de La Frontera, Temuco 4780000, Chile; 7 Department of Internal Medicine, Faculty of Medicine, Universidad de La Frontera, Temuco 4780000, Chile; 8 Institute of Parasitology, Justus Liebig University Giessen, 35392 Giessen, Germany; carlos.r.hermosilla@vetmed.uni-giessen.de (C.H.); anja.taubert@vetmed.uni-giessen.de (A.T.)

**Keywords:** PMN, NETosis, spermatozoa, oxidative stress, antioxidants

## Abstract

In swine reproduction, immune-mediated mechanisms such as neutrophil extracellular trap (NET) formation can affect sperm function and reduce fertility outcomes. This study evaluated the capacity of antioxidant and reproductive compounds—butylated hydroxytoluene (BHT), prostaglandin E_2_ (PGE_2_), bovine serum albumin (BSA), and seminal plasma (SP)—to modulate NETosis in co-cultures of swine neutrophils and cryopreserved spermatozoa. NET formation was quantified by nuclear area expansion and validated by digital cytometry and immunofluorescence. BHT (0.5 mM) and PGE_2_ (10 µM) produced the most significant inhibitory effects, reducing NETotic cell percentages from 34.5 ± 2.7% (sperm-exposed controls) to 12.2 ± 1.3% and 14.5 ± 2.1%, respectively (*p* < 0.01). SP at 20% decreased NETosis to 16.8 ± 1.8%, while BSA (0.5%) achieved a moderate reduction to 21.3 ± 2.5%. Flow cytometry revealed reduced peroxynitrite levels in sperm treated with SP and BSA. Two NET phenotypes (*agg*NETs and *spr*NETs) were identified. BTS medium enhanced NET formation, whereas DNase I degraded NETs effectively. These findings identify porcine NETosis as a redox-sensitive pathway modulated in vitro, suggesting an immunological role in enhancing sperm preservation for swine artificial insemination.

## 1. Introduction

In swine reproduction, biotechnological advances have prompted the use of low-dose artificial insemination (AI) protocols. However, conventional AI techniques have failed to yield satisfactory fertility outcomes under these conditions [1]. Interestingly, higher pregnancy rates were obtained by deposition near the oviduct [2]. This disparity suggests that the uterine environment may act as a critical immunological checkpoint influencing sperm survival and fertilization efficiency [3]. Given the well-documented presence of innate immune components, including neutrophils and pattern recognition receptors within the uterus, it is likely that immune-mediated mechanisms, such as sperm phagocytosis, inflammatory signaling, or extracellular trap formation, play a pivotal role in modulating sperm transit and function [4]. Therefore, a deeper understanding of the immunological landscape of the porcine female reproductive tract, and its interaction with exogenous spermatozoa, is essential to improve the success of AI strategies [2].

Neutrophil extracellular traps (NETs) are intricate networks of decondensed chromatin and antimicrobial proteins, such as neutrophil elastase (NE) and myeloperoxidase (MPO), released in response to diverse stimuli [5,6,7], including the presence of spermatozoa [8]. Their formation through the classical pathway critically depends on reactive oxygen species (ROS) generated by NADPH oxidase (NOX) [9]. This process, known as NETosis, plays a crucial role in the innate immune response, helping to eliminate pathogens and foreign particles [10]. However, in the context of swine reproduction, NETs can also entrap and impair spermatozoa, potentially affecting fertility by compromising sperm motility and integrity [8]. Research has focused on understanding interactions between neutrophils and spermatozoa, particularly on how these complex interactions might influence reproductive outcomes [11]. As such, viable spermatozoa of different mammalian species can induce NETosis in exposed neutrophils, leading to the formation of NETs that can capture and damage sperm [11]. NETs exhibit distinct morphological types: aggregated NETs (*agg*NETs) are dense, clustered structures formed by multiple neutrophils; spread NETs (*spr*NETs) are thin, filamentous, and widely spread; and diffused NETs (*diff*NETs) represent diffuse chromatin clouds with less structure, typically associated with early or partial NET release [12]. Swine spermatozoa have been shown to trigger the formation of *agg*NETs, which can effectively entrap numerous sperm at once, thereby leading to a decrease in motility and membrane integrity [8,13].

In vitro neutrophil–sperm co-cultures offer a controlled model to study NETosis, which rapidly entrap spermatozoa within 1–3 h in bovine and porcine polymorphonuclear neutrophils (PMN) [8,11]. Several antioxidant candidates are known as NETosis modulators or inhibitors in other mammalian species [14,15,16,17,18,19,20]. Albumin, a major plasma protein, is known for its antioxidant properties, and exhibited ROS scavenging activity and thus inhibited NETs in human and murine models [21,22]. In addition, butylated hydroxytoluene (BHT), a lipophilic antioxidant, is recognized for its ability to neutralize ROS, by interrupting the signaling cascade necessary for chromatin decondensation and NET release in mice [23]. Additionally, prostaglandin E_2_ (PGE_2_), a bioactive lipid mediator abundant in semen and inflamed tissues, has been shown to inhibit NET formation induced by phorbol myristate acetate (PMA) or ionomycin in both human and murine PMN [24,25]. Finally, seminal plasma, the fluid fraction of semen, contains numerous immunomodulatory components like spermadhesin (PSP-I/PSP-II) that actively induce neutrophil migration into the uterus [26]; notably, seminal plasma harbors nucleases like DNase that can degrade extracellular DNA traps, potentially freeing sperm from NETs [27]. Seminal plasma also carries inflammatory factors (e.g., prostaglandins, cytokines) that may influence PMN responses in the female tract [28].

Despite these insights, a clear gap remains regarding how NETosis modulators affect neutrophil–sperm dynamics in swine, especially in cryopreserved sperm, which often exhibit oxidative alterations that may amplify inflammatory responses and reduce fertility [29]. This study aims to investigate the effects of albumin, BHT, PGE_2_, and seminal plasma on NETotic cell activation in swine neutrophil–spermatozoa co-cultures in vitro in terms of PMN activation and NETotic release. By exploring these interactions, we seek to understand how these compounds can be used to enhance porcine sperm quality and fertility outcomes by modulating the immune response in the reproductive tract.

## 2. Materials and Methods

### 2.1. Ethical Declaration

All experimental procedures and protocols described herein were reviewed and approved by the Scientific Ethics Committee of the Universidad de La Frontera (UFRO), Temuco, Chile, under authorization code N°121/23 (approval date 10 May 2023). All procedures complied with the provisions of the Chilean Animal Protection Act (Law N° 20,380). The blood and semen samples were obtained from Agricultural and Livestock Society Pehuén Ltd.a. (Victoria, Chile) under standard breeding conditions.

### 2.2. Collection and Isolation of Polymorphonuclear Neutrophils (PMN)

PMN were isolated from venous whole blood collected from three healthy sows (n = 3; female pigs were housed at Agricultural and Livestock Society Pehuén Ltd.a. in Victoria, Chile), following a modified protocol [30]. Briefly, 10 mL of freshly drawn blood was mixed with 10 mL of phosphate-buffered saline (PBS) supplemented with EDTA (0.1488 g/200 mL; pH 6.8). The suspension was centrifuged at 1000× *g* for 15 min at room temperature (RT), without a break. Following centrifugation, the plasma, leukocyte layer, and upper erythrocyte layer were carefully discarded. A 2 mL fraction of the remaining erythrocyte-rich layer was resuspended in 2 mL of PBS and subjected to hypotonic lysis by the addition of 12 mL of distilled water. After 45 s, isotonicity was restored by adding 6 mL of 2.7% NaCl in PBS. The suspension was then centrifuged at 125× *g* for 10 min at RT. The supernatant was discarded, leaving approximately 2 mL of residual volume. The hypotonic lysis step was repeated once more to ensure complete removal of red blood cells. After the final centrifugation, the pellet was resuspended in Hank’s Balanced Salt Solution (HBSS) without calcium and magnesium for immediate use in functional assays. Cell count and viability were assessed by trypan blue exclusion using the Countess 3 Cell Counter^®^ (Thermo Fisher Scientific, Waltham, MA, USA). Only PMN populations with confirmed viability greater than 95% were used for subsequent experiments.

### 2.3. Semen Collection, Sperm Preparation, and Cryopreservation

Three ejaculates were collected from each of eight mature Landrace boars with documented field fertility. Semen was collected using the gloved-hand technique, excluding the gelatinous fraction, and deposited into pre-warmed collection vessels covered with sterile gauze.

Immediately following collection, the sperm-rich fraction was diluted in AndroStar Plus^®^ extender (Minitüb, Tiefenbach, Germany), and commercial semen doses were prepared, each containing 80 mL with a final concentration of 30 × 10^6^ sperm/mL. All semen samples were maintained at 17 °C and transported to the laboratory within one hour post-collection. To minimize experimental variability, semen doses were stored in a refrigerated chamber at 17 °C for 24 h prior to use. Regarding the pre-freeze quality criteria (≥90% total motility and ≥80% viability), those analyses were performed through visual assessment based on expert judgment, and only post-thaw sperm samples exhibiting ≥50% motility and ≥50% viability were included in the experimental procedures. These post-thaw thresholds were used to ensure a consistent functional baseline for downstream co-culture and flow cytometry assays [31].

Cryopreservation was performed according to the method described before [32]. After a 24 h holding period at 17 °C, semen samples were centrifuged at 800× *g* for 10 min in a temperature-controlled centrifuge set to 17 °C. The resulting sperm pellet was resuspended in extender A to a concentration of 1.5 × 10^9^ sperm/mL and then cooled to 5 °C over a period of 120 min. Subsequently, extender B (composed of extender A supplemented with glycerol and Equex^®^, Nova Chemical Sales, Inc., Scituate, MA, USA) was added to adjust the final sperm concentration to 1 × 10^9^ sperm/mL. The extended semen was then loaded into 0.25 mL plastic straws. Freezing was performed using an automated programmable freezer (IceCube 11 XS, Minitüb) with the following temperature descent curve: −6 °C/min for 100 s (from 5 to −5 °C); −39.82 °C/min for 113 s (from −5 to −80 °C); a hold at −80 °C for 30 s; followed by −60 °C/min for 70 s (from −80 to −150 °C). Frozen straws were stored in liquid nitrogen tanks until use. Thawing was conducted by immersing the straws in a thermo-regulated water bath at 38 °C for 20 s, followed by dilution in Beltsville Thawing Solution (BTS) at a 1:4 ratio (semen/ BTS).

### 2.4. Co-Culture Conditions and Treatments

**Figure 1:**
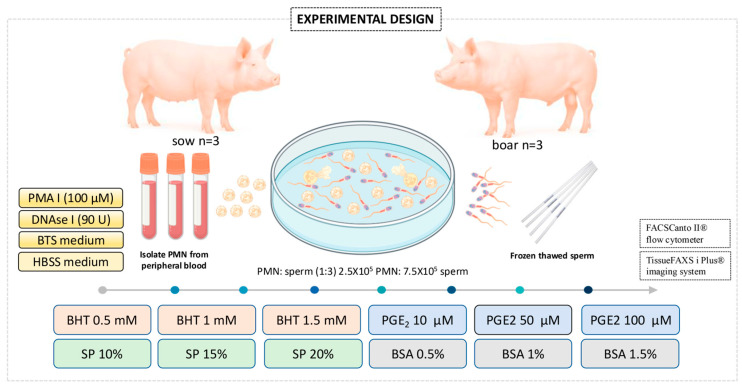
Experimental design for evaluating the modulation of NETosis in swine neutrophil–sperm co-cultures. Polymorphonuclear neutrophils (PMN) and spermatozoa were co-incubated under different treatments, including BHT, PGE_2_, SP, and BSA, using HBSS or BTS as culture media.

The aim of this study was to investigate the effects of the different antioxidant compounds on sperm-triggered NETs and their potential use in porcine reproduction. An in vitro co-culture model was established using PMN and spermatozoa. Co-cultures were performed in 8-well flat-bottom plates by combining 2.5 × 10^5^ PMN with 7.5 × 10^5^ spermatozoa (1:3 ratio) in a final volume of 200 µL [8]. The cell suspensions were incubated at 38 °C for 60 min under various antioxidant treatment conditions, as illustrated in Figure 1. All the following treatments were added simultaneously with spermatozoa at the start of the 60 min co-culture period: BHT at 0.5, 1.0, and 1.5 mM; PGE_2_ at 10, 50, and 100 µM; reproductive components: SP at 10%, 15%, and 20%; control protein supplementation: BSA at 0.5%, 1.0%, and 1.5%. The concentrations selected for each compound in this study were determined based on preliminary dose–response experiments, which confirmed that these ranges produced measurable effects on NETosis modulation without inducing cytotoxicity. Each treatment was prepared in Hank’s Balanced Salt Solution (HBSS). HBSS and BTS were evaluated as medium experimental groups. PMA (100 µM) was used as a positive control for porcine NETosis induction, while DNase I (90 U/mL) was used to degrade extracellular DNA structures (negative control). Following incubation, cells were processed for NETosis quantification.

### 2.5. Detection and Quantification of NETotic Cells

The percentage of NETotic neutrophils was determined by quantifying nuclear area expansion (NAE), a validated morphological hallmark of NETosis [11]. Nuclear segmentation was performed using an epifluorescence-based digital cytometry approach, as described previously [11], and implemented with StrataQuest^®^ analysis software (v. 7.0, TissueGnostics, Vienna, Austria) [11]. For each condition, samples were stained with Sytox Orange^®^ (Invitrogen, Thermo Fisher Scientific, Waltham, MA, USA), a DNA-binding dye. Nuclear masks were automatically generated to segment individual nuclei based on fluorescence intensity (FI) thresholds. These thresholds were manually adjusted to exclude non-neutrophilic signals such as spermatozoa and cellular debris. Within each image, a minimum of 2 × 10^4^ nuclei were analyzed. The area occupied by each nucleus was quantified in µm^2^. PMN with NAE exceeding 60 µm^2^ were operationally defined as NETotic cells, in accordance with previously established criteria for NAE-based NETosis detection and quantification [11]. The 60 µm^2^ threshold was determined empirically using control pig PMN that were viable, non-activated, and cultured under baseline conditions without stimulants. This threshold was applied uniformly across all treatment groups. For each experimental condition, a minimum of 2 × 10^4^ nuclei were analyzed within a 5 mm^2^ image area per well. Fluorescent signals were captured from samples stained with Sytox Orange^®^, and the fluorescence intensity threshold was manually calibrated to eliminate interference from sperm nuclei and cellular debris. The nuclear area of each segmented PMN was quantified in µm^2^, and the proportion of NETotic cells (nuclei > 60 µm^2^) was calculated for each sample.

### 2.6. Immunofluorescence and NET Quantification

PMN were co-incubated with porcine spermatozoa at a 1:3 ratio (2.5 × 10^5^ PMN/mL: 7.5 × 10^5^ sperm/mL) for 60 min at 38 °C in a humidified atmosphere containing 5% CO_2_. Following incubation, cells were fixed with 4% paraformaldehyde for 15 min at RT, then washed with sterile phosphate-buffered saline (PBS) and blocked in PBS supplemented with 2% bovine serum albumin (BSA) for 30 min at room temperature. For primary immunolabeling, samples were incubated overnight (15 h) at RT with a rabbit polyclonal anti-neutrophil elastase (NE) antibody (Abcam, Cambridge, UK) diluted 1:300 in PBS containing 2% BSA. After primary incubation, samples were washed three times with 200 µL of PBS under gentle agitation (100 rpm, 5 min per wash), and subsequently incubated for 1 h at room temperature—protected from light—with a goat anti-rabbit IgG secondary antibody conjugated to Alexa Fluor™ 488 (Invitrogen, Thermo Fisher Scientific, Waltham, MA, USA), diluted 1:500 in PBS with 2% BSA [8]. Finally, samples were counterstained with Sytox Orange^®^ for nuclear visualization and mounted using antifade reagent. Fluorescence images were acquired using the TissueFAXS i Plus^®^ imaging system (TissueGnostics, Vienna, Austria) equipped with a 20× objective. NETs were identified and quantified by the co-localization of extracellular NE and DNA.

### 2.7. Oxidative Stress and Sperm Viability Assays in Flow Cytometry

To evaluate oxidative stress markers and sperm viability, a series of flow cytometry assays were conducted on frozen–thawed sperm samples incubated with BHT, PGE_2_, SP, and BSA, respectively. All procedures were performed using a FACSCanto II^®^ flow cytometer (Becton Dickinson and Company, BD Biosciences, San Jose, CA, USA).

Cholesterol redistribution assessment was assessed using Merocyanine-540 (M-540 (Thermo Fisher Scientific, Waltham, MA, USA) in conjunction with SYTOX Green^®^ to exclude non-viable cells. A total of 2 × 10^6^ frozen–thawed spermatozoa were suspended in 500 µL of Beltsville Thawing Solution (BTS) containing M-540 at a final concentration of 250 µM, SYTOX Green^®^ (Molecular Probes, Eugene, OR, USA) at 0.04 µM, and Hoechst dye. The suspension was incubated at 38 °C for 20 min. Post-incubation, samples were centrifuged at 800× *g* for 5 min, and the pellet was resuspended in 500 µL of BTS. Flow cytometric analysis focused on quantifying the percentage of viable spermatozoa exhibiting low M-540 fluorescence (SYTOX^−^/M-540^−^), indicative of intact membrane lipid order. SYTOX and M-540 were simultaneously excited using a 488 nm blue laser, with fluorescence emissions collected using 530/30 nm and 585/42 nm bandpass filters, respectively.

Membrane lipid peroxidation levels were determined using the fluorescent probe C11-BODIPY581/591 (Invitrogen, Thermo Fisher Scientific, Waltham, MA, USA). In this assay, 2 × 10^6^ frozen–thawed spermatozoa were incubated in 400 µL of BTS supplemented with Hoechst dye and C11-BODIPY581/591 at a final concentration of 5 μM at 38 °C for 30 min. Following incubation, samples were centrifuged at 800× *g* for 5 min, and the pellet was resuspended in 500 µL of BTS. Flow cytometry was employed to measure the mean fluorescence intensity (MFI) of C11-BODIPY581/591 within the total sperm population (Hoechst^+^), reflecting the extent of lipid peroxidation. Emission from the reduced form was detected using a 585/42 nm bandpass filter (orange/red channel), while the oxidized form was detected with a 530/30 nm bandpass filter (green channel). The ratio of oxidized to reduced fluorescence within the Hoechst^+^ sperm population was quantified to determine the extent of lipid peroxidation.

To simultaneously evaluate mitochondrial membrane potential (MMP) and peroxynitrite levels, sperm samples were stained with tetramethylrhodamine methyl ester (TMRM) and dihydrorhodamine 123 (DHR), respectively. A suspension of 2 × 10^6^ frozen–thawed spermatozoa in 1 mL of BTS was prepared, to which TMRM (final concentration: 250 µM), DHR (1 µM), and propidium iodide (PI; 12 µM) were added. The mixture was incubated at 38 °C for 20 min. Post-incubation, samples were centrifuged at 800× *g* for 5 min, and the pellet was resuspended in 500 µL of BTS. Flow cytometric analysis was conducted to determine the MFI of TMRM and DHR within the viable sperm population (PI^−^), providing insights into mitochondrial function and oxidative stress levels. Both probes were excited using a 488 nm blue laser. Emission from TMRM, indicative of intact mitochondrial potential, was collected using a 585/42 nm bandpass filter, while oxidized DHR fluorescence, reflecting peroxynitrite generation, was detected with a 530/30 nm bandpass filter. Propidium iodide (PI) was used to exclude non-viable cells (detected with a 610/20 nm filter), and analysis was restricted to the viable sperm population (PI^−^).

Regarding gating strategies used for viability assessment, sperm populations were first identified by forward and side scatter (FSC/SSC) and further gated based on Hoechst positivity to exclude debris. Propidium iodide (PI) was used to distinguish viable (PI^−^) from non-viable (PI^+^) sperm. Only PI^−^/Hoechst^+^ events were considered viable and included in the analysis of mitochondrial membrane potential (TMRM) and peroxynitrite levels (DHR). For oxidative stress assays involving SYTOX Green and Merocyanine-540 (M-540), a similar strategy was applied: viable sperm were defined as SYTOX Green^−^/M-540^−^ within the Hoechst^+^ population.

All experimental conditions were evaluated in three independent biological replicates, each using spermatozoa from different boars and neutrophils from distinct sows. Additionally, technical triplicates were performed within each replicate to ensure statistical robustness and reproducibility.

### 2.8. Statistical Analysis

All flow cytometry data, including the quantification of NETotic cells, were subjected to logarithmic transformation to normalize distributions and stabilize variances. Subsequently, a one-way analysis of variance (ANOVA) was employed to assess differences among experimental groups. When the ANOVA indicated significant differences (*p* < 0.05), Tukey’s Honest Significant Difference (HSD) post hoc test was conducted to perform pairwise comparisons between group means. Statistical significance was determined at a threshold of *p* < 0.05. All analyses were performed using GraphPad Prism^®^ software v. 10.1.1.

## 3. Results

### 3.1. Antioxidant and Reproductive Compounds Inhibit Sperm-Induced NETosis In Vitro

**Figure 2:**
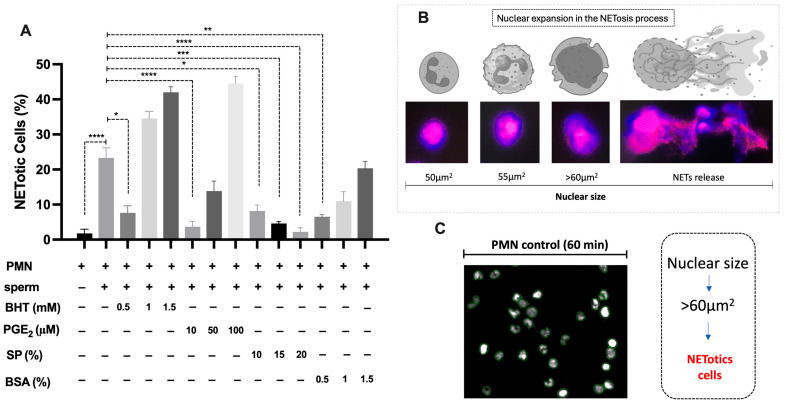
Inhibition of sperm-induced NETosis by antioxidant and reproductive compounds in vitro. (**A**) Quantification of NETotic cells (%) after 60 min of co-incubation of PMN with spermatozoa in the presence or absence of BHT, PGE_2_, SP, and BSA. The proportion of NETotic cells was determined based on nuclear area expansion (NAE) exceeding 60 µm^2^, assessed by fluorescence microscopy and image analysis. Data are expressed as mean ± SD from independent replicates (n = 3). Statistical significance (* *p* < 0.05, ** *p* < 0.01, *** *p* < 0.001, **** *p* < 0.0001). (**B**) Schematic representation and microscopic validation of nuclear morphological changes associated with NETosis. Progressive NAE was observed from early chromatin decondensation (~50–55 µm^2^) before NET release (>60 µm^2^). Fluorescent staining (DNA in magenta) demonstrates nuclear size thresholds used to classify cells as NETotic. (**C**) Representative fluorescence image of PMN under control conditions; this threshold was applied consistently for quantitative analysis.

The co-incubation of swine PMN with spermatozoa for 60 min resulted in a significant increase in the proportion of NETotic cells, as determined by NAE (Figure 2A). In contrast, treatment with BHT, PGE_2_, and BSA markedly reduced NETosis rates compared to the sperm-exposed PMN without treatment.

Among the tested compounds, BHT demonstrated a dose-dependent inhibitory effect, with 0.5 mM achieving the greatest reduction in NETotic cell percentage. Similarly, PGE_2_ treatment at 10 µM significantly suppressed NET formation. SP also exhibited a concentration-dependent inhibition of NETosis, with 20% SP producing the most pronounced effect. BSA supplementation at 0.5% reduced NETotic cell proportions.

The classification of NETotic cells was based on fluorescence microscopy and digital image analysis, applying a consistent nuclear size threshold of >60 µm^2^ across all experimental groups (Figure 2B,C). Progressive nuclear morphological changes associated with NETosis were validated, including early chromatin decondensation (~50–55 µm^2^), leading to the evaluation of NETotic cells before extracellular trap release (>60 µm^2^). Control PMN maintained nuclear integrity with minimal expansion, supporting the validity of the analytical threshold here applied.

### 3.2. Digital Cytometry Reveals Differential Nuclear Expansion Profiles During Sperm-Induced NETosis

**Figure 3:**
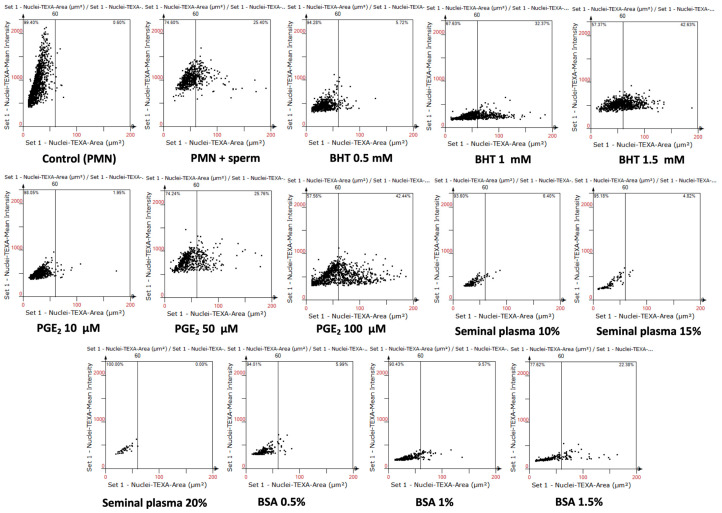
Cell-based scatter plots of nuclear expansion during swine sperm-induced NETosis, analyzed by digital cytometry using TissueFAXS i Plus and StrataQuest software v. 7.0. Scatter plots display nuclear area (µm^2^) versus nuclear DNA fluorescence intensity (TEXA channel) of individual neutrophils analyzed after 60 min of incubation. The vertical demarcation line at 60 µm^2^ indicates the threshold used to classify NETotic cells, based on nuclear area expansion (NAE), an established morphological hallmark of NETosis.

Cell-based scatter plot analysis demonstrated distinct patterns of NAE following co-incubation of swine PMN with spermatozoa for 60 min (Figure 3). In control PMN cultured alone, nuclear areas remained predominantly below the 60 µm^2^ threshold, indicating minimal spontaneous NETosis.

Exposure to spermatozoa markedly shifted the distribution, with a substantial proportion of nuclei expanding beyond 60 µm^2^, consistent with active NET formation. Treatment with BHT, PGE_2_, SP, or BSA effectively attenuated this expansion, as evidenced by a reduced density of events above the NETotic threshold in the corresponding scatter plots.

Specifically, BHT at 0.5 mM and PGE_2_ at 10 µM showed a pronounced inhibitory effect, characterized by a clustering of nuclei within smaller area ranges (<60 µm^2^) and decreased fluorescence intensity. Concentrations of SP (10–20%) also led to a marked reduction in nuclear expansion, suggesting potent modulation of sperm-induced NETosis. BSA-treated groups exhibited inhibitory effects in low concentrations similar to antioxidant and reproductive fluid treatments.

These cytometric profiles corroborate the quantitative findings obtained by fluorescence microscopy and validate the use of NAE as a reliable morphological biomarker to assess NET formation under different experimental conditions.

### 3.3. Medium Composition and Modulatory Treatments Influence Sperm-Induced NETosis in Swine PMN

**Figure 4:**
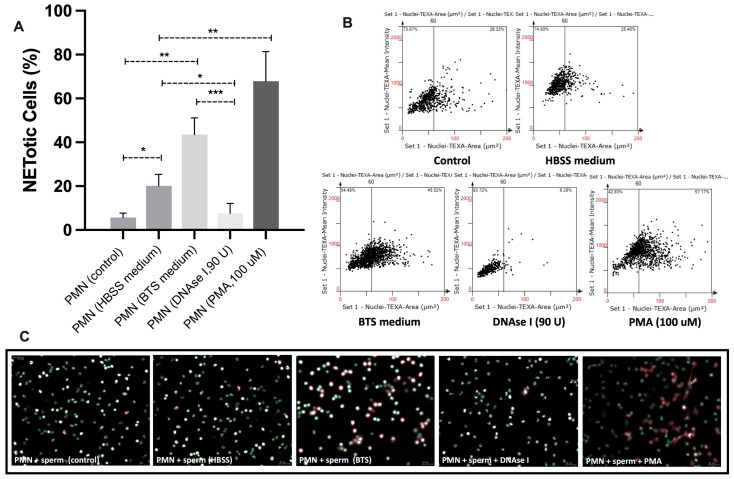
Evaluation of NETotic response in swine PMN under different medium and stimulation conditions. (**A**) Quantification of NETotic cells (%) after 60 min of incubation in HBSS and BTS medium, or in the presence of DNase I (90 U) or PMA (100 µM). NETosis was determined by the percentage of cells with nuclear area >60 µm^2^. Data are expressed as mean ± SD (* *p* < 0.05, ** *p* < 0.01, *** *p* < 0.001). (**B**) Cell-based scatter plots showing nuclear area versus DNA fluorescence intensity obtained via StrataQuest^®^ software from samples digitized with the TissueFAXS i Plus^®^ system. The vertical line at 60 µm^2^ indicates the threshold used to classify NETotic cells. (**C**) Representative fluorescence images of PMN co-incubated with spermatozoa under each treatment condition. DNA was stained with Sytox Orange^®^; expanded nuclei (>60 µm^2^) appear in red. Images were acquired with a 20× objective on the TissueFAXS i Plus platform. Scale bar = 20 µm.

The NETotic response of swine PMN was significantly influenced by the culture medium and by the presence of modulatory agents (Figure 4A). PMN maintained in HBSS exhibited an increase in NETotic cells compared to untreated controls, while incubation in BTS medium further enhanced NETosis levels. Conversely, treatment with DNase I (90 U) markedly reduced the percentage of NETotic cells, confirming the role of extracellular DNA structures in swine PMN-derived NET formation. As expected, PMA (100 µM) served as a strong positive control, inducing the highest levels of NETosis among the tested conditions.

Scatter plot analysis (Figure 4B) of nuclear area versus DNA fluorescence intensity revealed a clear shift in nuclear morphology under different conditions. While BTS and PMA treatments promoted a greater proportion of nuclei exceeding the 60 µm^2^ threshold, DNase I treatment preserved a nuclear profile similar to baseline conditions, with minimal nuclear expansion.

Representative fluorescence images (Figure 4C) corroborated these findings. In samples incubated with either BTS or PMA, a higher number of PMN with expanded nuclei (>60 µm^2^) were observed, visualized as red-stained structures. In contrast, DNase I-treated samples exhibited fewer NETotic cells, maintaining predominantly small and compact nuclei.

### 3.4. Quantification of NET Release Confirms Modulatory Effects of Antioxidant and Reproductive Compounds

**Figure 5:**
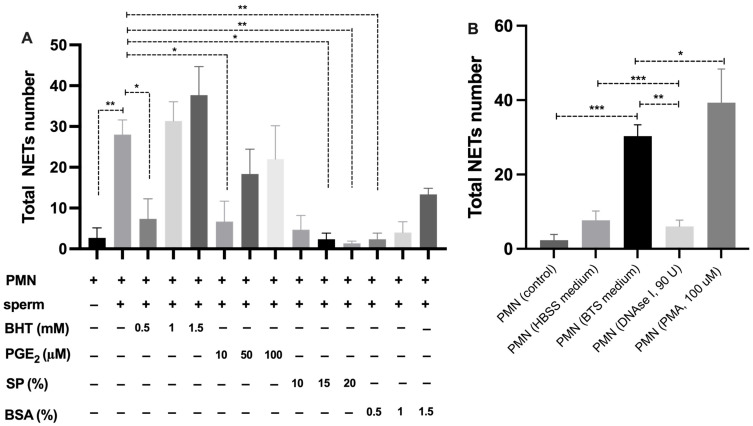
Quantification of NET released by swine PMN after co-incubation with spermatozoa under various treatment conditions. (**A**) Total number of NET detected per field after 60 min of incubation with BHT; 0.5–1.5 mM, PGE_2_; 10–100 µM, SP; 10–20%, or BSA; 0.5–1.5%. (**B**) Comparison of total NET released under different media and stimulatory conditions: control (PMN), HBSS, BTS medium, DNase I (90 U), and PMA (100 µM). NET were quantified by image analysis based on immunofluorescence labeling and morphological identification. Data are presented as mean ± SD. Statistical significance (* *p* < 0.05, ** *p* < 0.01, *** *p* < 0.001).

Quantification of NET structures revealed that co-incubation of swine PMN with spermatozoa under various treatment conditions resulted in differential NET release profiles (Figure 5). Analysis of total NET numbers per microscopic field showed that treatment with BHT, PGE_2_, SP, and BSA significantly modulated NET formation compared to sperm-stimulated controls (Figure 5A).

Among antioxidant treatments, BHT at 0.5 mM exhibited the most pronounced reduction in NET numbers. Similarly, PGE_2_ at a lower concentration (10 µM) substantially decreased NET release. Seminal plasma at 15–20% concentrations led to a marked suppression of NET formation, whereas BSA supplementation induced consistent decreases across the 0.5% tested concentration.

When comparing culture media and stimulation conditions (Figure 5B), the BTS medium promoted a higher NET release compared to the control group. As expected, PMA stimulation induced the greatest number of NET structures per field, while DNase I treatment significantly reduced NET counts, corroborating its capacity to degrade extracellular chromatin networks.

### 3.5. Swine PMN Release Distinct Morphological Forms of NET Following Sperm Stimulation

**Figure 6:**
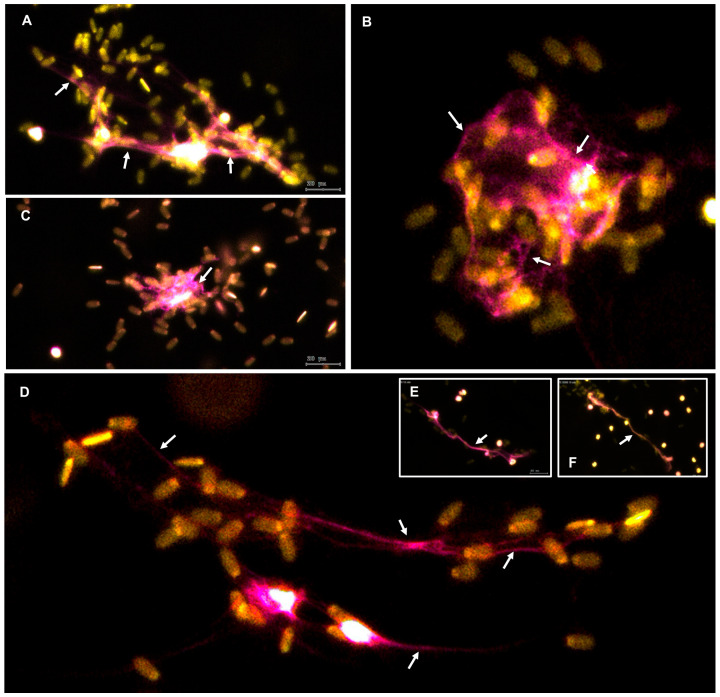
Representative merged immunofluorescence images showing distinct morphological forms of NET released by swine PMN after 1 h of incubation. Neutrophil elastase (magenta) was detected using an anti-NE antibody, and nuclear DNA was stained with DAPI (yellow). All images were acquired using the TissueFAXS i Plus imaging system with a 20× objective and are presented as merged fluorescence channels. (**Panels A**–**C**) display *agg*NET, characterized by dense chromatin structures surrounded by extracellular elastase. (**Panels D**–**F**) show elongated *spr*NET, defined by extended filamentous structures. NET are indicated by white arrows. Scale bar = 20 µm.

Immunofluorescence analysis revealed heterogeneous morphological patterns of NET structures released by swine PMN after 60 min of co-incubation with spermatozoa (Figure 6). Merged images of neutrophil elastase (NE, magenta) and nuclear DNA (Sytox Orange^®^, yellow) staining demonstrated two predominant NET phenotypes.

Panels A–C depict *agg*NET, characterized by dense clusters of decondensed chromatin surrounded by extracellular NE. These structures displayed a compact morphology with an intense fluorescent signal, indicating localized NET deployment around groups of entrapped spermatozoa.

In contrast, panels D–F illustrate elongated spread NETs (*spr*NET), presenting as filamentous, extended DNA–protein networks dispersed across the field. These elongated structures appeared to physically tether spermatozoa, potentially restricting their motility and interaction with surrounding cells. NET structures were consistently identified by the colocalization of extracellular NE with DNA signals and are highlighted by white arrows in the images.

### 3.6. Antioxidant and Reproductive Compounds Modulate Viability, Oxidative Stress, and Mitochondrial Function in Porcine Spermatozoa

**Figure 7:**
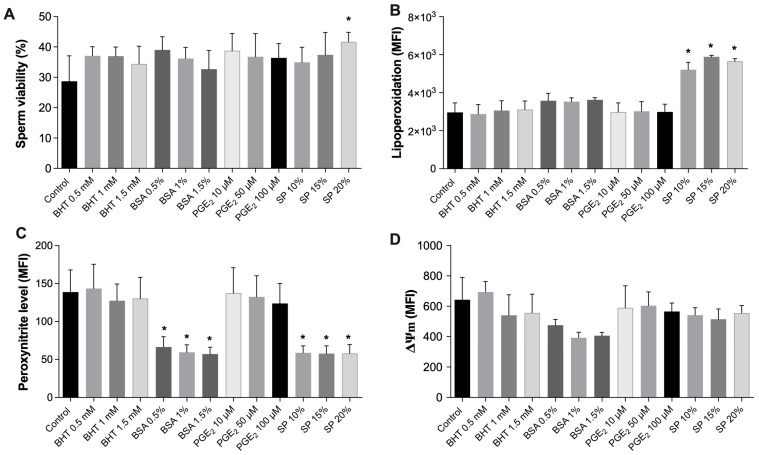
Porcine spermatozoa were incubated for 60 min with antioxidant and reproductive compounds to assess their effects on cell viability, oxidative stress, and mitochondrial function. Treatments included BHT; 0.5–1.5 mM, BSA; 0.5–1.5%, PGE_2_; 10–100 µM, and SP; 10–20%. (**A**) Sperm viability. (**B**) Lipid peroxidation. (**C**) Peroxynitrite levels. (**D**) Mitochondrial membrane potential (ΔΨm). Data are expressed as mean fluorescence intensity (MFI) or percentage of viable cells. Asterisks (*) indicate statistically significant differences compared to the control group (* *p* < 0.05).

The effects of antioxidant and reproductive compounds on sperm viability, oxidative stress, and mitochondrial function were assessed after 60 min of incubation (Figure 7). Sperm viability (Figure 7A) remained largely stable across treatments with BHT, BSA, and PGE_2_, while a significant increase in viability was observed in samples incubated with 20% SP.

Lipid peroxidation levels (Figure 7B) were significantly elevated in spermatozoa exposed to higher SP concentrations (15–20%), indicating increased oxidative membrane damage under these conditions. By contrast, BHT, BSA, and PGE_2_ treatments did not significantly alter lipid peroxidation compared to controls.

Peroxynitrite generation (Figure 7C) was significantly reduced following treatment with BSA at 0.5–1.5% and with SP at 10–20%, suggesting an effective antioxidant action in these conditions. BHT and PGE_2_ treatments showed modest but non-significant effects on peroxynitrite levels.

Mitochondrial membrane potential (ΔΨm) (Figure 7D) exhibited slight decreases across treatments; however, no statistically significant differences were detected compared to control spermatozoa, indicating that mitochondrial functionality remained largely preserved after exposure to the tested compounds.

## 4. Discussion

The results of this study indicate that swine PMN exhibited a pronounced NETotic response upon spermatozoa exposure, aligning with findings in humans and bovines where sperm triggered NET formation as part of a conserved innate defense mechanism in mammalian reproduction [11,33]. Characterized by chromatin–protein extrusion, this response leads to sperm entrapment and impaired motility [8]. We identified two NET subtypes, *spr*NETs and *agg*NETs, mirroring those described in other species [11], suggesting conserved morphological variants of NETosis with potential functional roles in reproductive immune regulation.

Critically, all tested compounds, BHT, PGE_2_, BSA, and seminal plasma, significantly reduced the percentage of NETotic cells. The antioxidant BHT was particularly effective, suggesting that ROS play a central role in sperm-induced NETosis [9]. Our results demonstrate that BHT at a concentration of 0.5 mM significantly inhibits sperm-induced NETosis in swine PMN co-cultures, a finding that aligns with its well-established role as a potent scavenger of ROS [34]. NET formation in response to spermatozoa appears to rely on ROS-dependent pathways, as previously shown in NETosis triggered by agents like PMA or monosodium urate (MSU) crystals [34]. BHT has been shown to suppress NETosis in such models by neutralizing ROS and disrupting the NOX–mediated oxidative burst necessary for chromatin decondensation and final NET release [35]. The reduction in NETotic cells in our study suggests that BHT acts upstream of nuclear breakdown, likely preventing the initiation of the NETotic cascade by quenching ROS, supporting the idea that sperm-induced NETosis in swine mirrors the classical, NOX-dependent pathway, given the sensitivity response to BHT inhibition. The pronounced inhibitory effect of BHT in NETosis confirms the redox-dependent nature of this immune response and underscores the therapeutic potential of antioxidants in preserving sperm functionality during post-insemination inflammation. Previous reports have indicated that excessive concentrations of BHT may undergo auto-oxidation, leading to a pro-oxidant shift that can promote rather than inhibit oxidative responses [36,37]. These effects appear to be species-dependent; for instance, murine neutrophils are particularly sensitive to the inhibitory action of BHT, whereas human neutrophils exhibit variable responses, likely due to differences in redox regulatory mechanisms [38]. This context may help explain the unexpected induction of NETosis observed in our model when using increasing concentrations of BHT.

On the other hand, PGE_2_ exerts a pronounced inhibitory effect on sperm-induced NETosis in swine PMN, corroborating mechanistic studies across species that indicate that PGE_2_ is a potent suppressor of NET formation through cAMP-dependent signaling pathways [24]. Specifically, PGE_2_ activates EP2 and EP4 receptors, both Gαs-coupled, which elevate intracellular cAMP levels and inhibit ROS production via downstream effectors such as protein kinase A (PKA) and Epac [24,25]. These mechanisms disrupt key processes required for NETosis, including NOX–mediated oxidative bursts and autophagy [25]. Although prior studies in humans and mice have shown that physiologically relevant concentrations of PGE_2_ of as low as 10 nM are sufficient to inhibit NET release, our results in swine neutrophils reveal that a higher concentration (i.e., 10 µM) is required to achieve a comparable suppressive effect. This difference may reflect species-specific sensitivity to PGE_2_ or variations in receptor density and signaling thresholds within neutrophil populations [25]. Therefore, our results suggest that PGE_2_ may play a critical regulatory role in reproductive immunity by attenuating excessive NET-mediated sperm entrapment through conserved molecular pathways, as observed in both immune-mediated diseases and therapeutic stem cell models [25,39]. Interestingly, low concentrations of PGE_2_ appear to inhibit NETosis, whereas higher concentrations promote its activation. This biphasic effect may be explained by the concentration-dependent engagement of distinct EP receptors. While low levels predominantly activate EP2 and EP4 receptors linked to cAMP-mediated anti-inflammatory pathways, higher concentrations may recruit EP1 or EP3 receptors, which are coupled to Gαq and Gαi proteins, respectively. Activation of these pathways can elevate intracellular Ca^2+^ levels, thereby promoting PAD4 activation and histone citrullination—critical steps in the initiation of NETosis [40]. The higher concentration of PGE_2_ required to inhibit NETosis in swine PMN, compared to human or murine models, may reflect species-specific differences in EP receptor subtype expression or signaling thresholds, although this remains to be confirmed experimentally.

Our findings reveal that porcine SP significantly suppresses NETosis in PMN–sperm co-cultures, aligning closely with results reported in horses, where SP mitigates PMN activation and reduces NET formation, thereby enhancing fertility by preserving sperm viability in inflamed uterine environments [41]. Nonetheless, this inhibitory role of SP contrasts sharply with that observed in donkeys and cattle. In donkeys, SP alone is sufficient to trigger NETosis in a time- and concentration-dependent manner, independent of sperm presence, suggesting a pro-inflammatory effect potentially linked to the low fertility rates seen with frozen–thawed semen [42]. Similarly, in cattle, SP components—particularly in semen supernatant—induce NETosis via Ca^2+^-dependent pathways involving NOX and PAD4, contributing to sperm entrapment and reduced fertilization efficiency [43]. The pronounced inhibitory effect of porcine SP observed in our swine model supports a species-specific immunomodulatory profile, resembling the equine rather than the bovine or donkey paradigm. This interspecies contrast, where SP exhibits anti-NETotic activity in pigs and horses, but pro-NETotic effects in cattle and donkeys, reveals a key knowledge gap regarding the divergent immunological roles of seminal plasma across species. These findings emphasize the need for further comparative studies to elucidate the mechanisms underlying SP–PMN interactions and their implications for post-insemination fertility outcomes. Few studies have evaluated the effect of the addition of SP during thawing; however, it has been reported that the proportion of viable spermatozoa with increased membrane fluidity (M540^+^) was considerably higher in samples incubated with 50% SP, which could be associated with an increase in ROS levels after 60 min of incubation [44]. The observation that SP increased lipid peroxidation in our experimental model warrants further consideration, as it contrasts with previous reports in the literature. Studies in equine models have demonstrated that the addition of seminal plasma to thawed semen does not significantly alter sperm membrane lipid peroxidation levels [45]. For instance, while seminal plasma reduced tyrosine phosphorylation on the surface of cryopreserved equine spermatozoa, it did not affect lipid peroxidation [45]. Similarly, it was found that post-thaw supplementation with seminal plasma preserved sperm viability and membrane stability without increasing oxidative damage [46]. The discrepancy between these findings and our results may reflect species-specific differences in seminal plasma composition, particularly in terms of antioxidant and pro-oxidant factors, or variations in experimental conditions such as sperm handling, extender composition, or cryopreservation protocols. While our findings support an anti-NETotic effect of porcine seminal plasma, direct experimental comparisons with bovine or donkey models were not performed in this study. Therefore, conclusions regarding species specificity should be interpreted with caution and warrant further cross-species investigation.

In our study, low concentrations of BSA effectively inhibited sperm-induced NETosis in swine neutrophils and significantly reduced peroxynitrite levels in sperm, suggesting a role for BSA in modulating redox-sensitive innate immune responses. These findings are consistent with reports in human neutrophils, where BSA suppresses NET formation by scavenging ROS and neutralizing NETosis inducers such as lipopolysaccharides (LPSs) and calcium ionophores [22]. The antioxidant effect of BSA is attributed to its free thiol groups, which become inactive upon oxidation, highlighting the importance of redox integrity in its inhibitory capacity [47]. Fluorescence anisotropy studies confirmed that BSA directly binds to LPS, providing a mechanistic basis for reduced NETosis under endotoxin exposure [22]. While BSA fails to inhibit PMA-induced NETosis in human cells due to its ROS-independent mechanism, it exerts broader inhibitory effects in murine models [22]. Our observation that BSA lowers peroxynitrite, a reactive nitrogen species associated with oxidative stress, in sperm supports the notion that its modulatory effect in swine is primarily redox-dependent. Interestingly, our results also showed that PMN cultured in BTS, the most commonly used medium for thawing cryopreserved boar spermatozoa that is enriched in glucose, exhibited increased NETosis activity, consistent with the role of glucose in promoting NOX2-dependent ROS bursts, which destabilize neutrophil membranes and promote chromatin decondensation [48].

As has been demonstrated in this study, both antioxidants and the composition of culture media can influence PMN activation and NETosis, as a redox-sensitive pathway that can be pharmacologically modulated with an effect on immunological responses in the female reproductive tract. This redox-sensitive pathway offers a promising immunological target to improve fertility outcomes in swine artificial insemination through the control of post-insemination inflammation.

## 5. Conclusions

This study provides in vitro evidence that porcine NET formation is a redox-sensitive process triggered by spermatozoa and significantly modulated by selected antioxidant and reproductive compounds. Among the treatments evaluated, butylated hydroxytoluene (BHT) at 0.5 mM was the most effective, reducing NETotic cell percentages from 34.5 ± 2.7% in sperm-exposed controls to 12.2 ± 1.3%. Prostaglandin E_2_ (PGE_2_) at 10 µM also showed a potent inhibitory effect, decreasing NETosis to 14.5 ± 2.1%. Seminal plasma at 20% reduced NET formation to 16.8 ± 1.8%, while bovine serum albumin (0.5%) achieved a moderate reduction to 21.3 ± 2.5%. These findings confirm that sperm-induced NETosis in swine is susceptible to pharmacological modulation through compounds with antioxidant or immunoregulatory properties. Additionally, the identification of two morphologically distinct NET phenotypes (*agg*NETs and *spr*NETs) and the influence of medium composition highlight the complexity of PMN–sperm interactions in vitro. Collectively, these findings underscore the therapeutic potential of modulating NETosis to improve sperm function and fertility outcomes in assisted reproduction. This work lays the groundwork for novel immunoregulatory strategies aimed at optimizing reproductive success through the control of post-insemination inflammation.

## Data Availability

The data supporting the findings of this study are available from the corresponding author upon reasonable request.
